# Low level laser (LLL) attenuate LPS-induced inflammatory responses in mesenchymal stem cells via the suppression of NF-κB signaling pathway in vitro

**DOI:** 10.1371/journal.pone.0179175

**Published:** 2017-06-08

**Authors:** Kan Yin, Rongjia Zhu, Shihua Wang, Robert Chunhua Zhao

**Affiliations:** Centre of Excellence in Tissue Engineering, Chinese Academy of Medical Sciences and Peking Union Medical College, Beijing, People's Republic of China; Univerzitet u Beogradu, SERBIA

## Abstract

**Background:**

Considering promising results in animal models and patients, therapeutic use of MSCs for immune disease is likely to undergo continued evaluation. Low-lever laser (LLL) has been widely applied to retard the inflammatory reaction. LLL treatment can potentially be applied in anti-inflammatory therapy followed by stem cell therapy.

**Aim of the study:**

The purpose of this study was to investigate the effect of LLL (660 nm) on the inflammatory reaction induced by LPS in human adipose derived mesenchymal stem cells (hADSCs) and pertinent mechanism.

**Materials and methods:**

Anti-inflammatory activity of LLL was investigated by LPS-induced mesenchymal stem cells. The production and expression of pro-inflammatory cytokines were evaluated by ELISA kits and RT-qPCR. Nuclear translocation of NF-κB was indicated by immunofluorescent staining. Phosphorylation status of NF-κB p65 and IκBα were illustrated by western blot assay. ROS generation was measured with CM-H2DCFDA, and NO secretion was determined by DAF-FM. We studied surface expression of lymphocyte activation markers when Purified peripheral blood mononuclear cell (PBMC) were activated by phytohaemagglutinin (PHA) in the presence of 3 types of treated MSCs.

**Results:**

LLL reduced the secretion of IL-1β, IL-6, IL8, ROS and NO in LPS treated MSCs. Immunofluorescent assay demonstrated the nuclear translocation decrease of NF-κB in LLL treated LPS induced MSCs. Western blot analysis also suggested that LLL suppressed NF-κB activation via regulating the phosphorylation of p65 and IκBα. MSC significantly reduced the expression of activation markers CD25 and CD69 on PHA-stimulated lymphocytes.

**Conclusion:**

The results indicate that LLL suppressed the activation of NF-κB signaling pathway in LPS treated MSCs through inhibiting phosphorylation of p65 and IκBα, which results in good anti-inflammatory effect. In addition, LLL attenuated activation-associated markers CD25 and CD69 in co-cultures of PBMC and 3 types of treated MSCs.

## Introduction

Low-level laser (LLL) used light with wavelengths in the range of 600nm to 1000nm and a power density between 1mW to 500 mW. It has a beneficial therapeutic effect for numerous diseases like Alzheimer’s Disease[1], multiple sclerosis[2] and temporomandibular joint disorders[3]. The mechanism for LLL was its stimulating of cytochrome c oxidase, thereby increasing mitochondrial activity and activating cell signaling cascades[4]. In recent years, the LLL has become widely recognized in the field of regenerative medicine[5].

Mesenchymal stem cells (MSCs) were first discovery by Alexander Friedenstein in the late 1960s. Therapeutic use of MSCs for regenerating medicine showed promising results in patients. The mechanisms of MSCs therapy include: paracrine activity like secretion of proteins; transfer of organelles by tunneling nanotubes; transfer molecules through exosomes[6]. MSCs are polarized by downstream TLR signaling into MSC1 and MSC2 phenotypes: TLR4 agonists polarized MSCs toward a pro-inflammatory MSC1 phenotype, whereas TLR3 stimulation of MSCs was toward an anti-inflammatory MSC2 phenotype[7]. The immunomodulatory capacity of MSCs is critical for their use in therapeutic applications[8].

Low lever laser irradiation has been shown to induce mesenchymal stem cells activity by increasing proliferation, migration and viability, activating protein expression and inducing differentiation in progenitor cells[9, 10]. The combination of bone marrow aspirate/LLL yielded significantly greater bone formation in surgically created critical-size defects in rat calvaria[11]. LLL was applied as an adjunct therapy for MSCs transplantation on the functional recovery of crushed sciatic nerve in rats[12]. MSCs were stimulated by LLL in order to affect neurological behavior and beta-amyloid burden in progressive stages of Alzheimer’s disease mouse model[13]. It has also been reported that LLL suppresses inflammatory response of human adipose-derived stem cells by modulating intracellular cyclic AMP level and NF-κB activity[14]. LLL is a valid approach for the preconditioning of MSCs in vitro prior cell transplantation[5]. However, the exact mechanism of action of LLL is not completely demonstrated and proved yet. The purpose of this study was to evaluate the effect of LLL on the LPS-induced inflammation response of hADSCs and pertinent cell signaling.

## Materials and methods

### Cells culture and treatment

Human adipose tissues were obtained with informed verbal consent and all experiments were approved by the Ethics Committee at the Chinese Academy of Medical Sciences and Peking Union Medical College, and all clinical investigations have been conducted according to the principles expressed in the Declaration of Helsinki. Adipose tissues were obtained from patients undergoing tumescent liposuction. AD-MSCs were isolated and culture expanded as previously reported. Passage 3 cells were used for the experiments[15]. For stimulated group, LPS (10 ng/mL, Sigma-Aldrich, St. Louis, MA, USA) was used as the agonists for TLR4 and incubated with the cells for 1 hr. Then the cells are washed twice in growth medium without the TLR4-agonist.

### Low lever laser irradiation

The exposure system used in the present experiment was designed by the medical apparatus company (No. LXW660-II, Jixing, Shenyang, China). The working conditions of the therapeutic apparatus: power source 220V±22V, 50Hz±1Hz; light output wavelength 660nm±20nm in the red to near infrared range (630–1000 nm), single point light output power 3mw-4.5mw, irradiance was 3w/m^2^-4.5w/m^2^, cells were irradiated for 1h to achieve energy density of 11–16 J/cm^2^. The laser beam was delivered using an optical fiber, and irradiated a circular area of 1cm^2^. All irradiation experiments were performed on a clean bench at room temperature. The control groups were processed under the same conditions, except without laser irradiation.

### Real-time RT-PCR

PCR primers specific to TLR-3 and to TLR-4 are designed to detect total RNA in AD-MSCs with the respective treatment. Real-time RT-PCR was performed to evaluate the expression of MSC1- and MSC2-related factors. IL-1ß, IL-6 and IL-8 were examined as MSC1-related factors while IL-4, IL-10 and IL-13 were examined as MSC2-related factors. Mitochondrial biogenesis related markers were also evaluated by real-time RT-PCR. Relative expression of mRNA was evaluated by 2^−ΔΔCt^ method and normalized to the expression of GAPDH. Primers used in this study for PCR were shown in Table 1.

**Table 1:** Primers used in study for PCR.

Gene	Forward	Reverse
**TLR-4**	5-AGACCTGTCCCTGAACCCTAT-3	5-CGATGGACTTCTAAACCAGCCA-3
**IL-1ß**	5-CTTCGAGGCACAAGGCACAA-3	5-TTCACTGGCGAGCTCAGGTA-3
**IL-6**	5CTCAATATTAGAGTCTCAACCCCCA3	5-GAGAAGGCAACTGGACCGAA-3
**IL-8**	5-CCACCGGAGCACTCCATAAG-3	5-GATGGTTCCTTCCGGTGGTT-3
**IL-4**	5-CTTTGCTGCCTCCAAGAACAC-3	5-GCGAGTGTCCTTCTCATGGT-3
**IL-10**	5-TTCCAGTGTCTCGGAGGGAT-3	5-GCTGGCCACAGCTTTCAAGA-3
**IL-13**	5-CCTATGCATCCGCTCCTCAA-3	5-AGCAATGACCGTGGTCAACA-3
**GAPDH**	5CCATGTTCGTCATGGGTGTGAACCA3	5GCCAGTAGAGGCAGGGATGATGTTC3

### Enzyme-linked immunosorbent assay (ELISA)

Cytokines were measured from cell culture media about 5ml in 6cm Petri dish with 5×10^6^ cells using commercial enzyme-linked immunosorbent assay (ELISA) kits from R&D according to manufacturer's instructions. Levels of IL-1ß, IL-6, IL-8, IL-4, IL-10, IL-13, in the supernatant of LLL or LPS treated MSCs were quantified.

### Immunofluorescence staining

For the detection of intracellular location of NF-κB p65 subunit, MSCs were seeded on confocal chamber. After 1 h of experiment, the cells were fixed in cold 4% paraformaldehyde, membrane-permeabilized by exposure to 0.2% Triton X-100 in cold PBS for 30 min, and blocked in 5% bovine serum albumin (BSA; in PBST [0.2% Tween-20 in PBS]) at room temperature for 30 min. The rabbit anti-NF-κB p65 subunit (1:200, diluted in PBST containing 5% BSA) was then used as the primary antibody and incubated with the cells for 2 h at room temperature. After sufficient washes with PBST, cells were incubated with FITC-labeled goat anti-rabbit IgG antibody (10 μg mL^−1^ diluted in PBST containing 5% BSA) for 1 h at room temperature in a dark place, and washed with PBST for 10 min. Cells were then stained with 5 μg mL^−1^ of DAPI for 30 min at 37°C in a dark place, followed by sufficient washes with PBS. Stained cells were analyzed using a Olympus FV1000 confocal microscope (Olympus, Tokyo, Japan), excitation wavelength 490 nm and emission wavelength 540 nm for FITC, excitation wavelength 360 nm and emission wavelength 450 nm for DAPI. Images were analyzed using FV10-ASW 4.0 Viewer software (Olympus).

### Measurement of intracellular reactive oxygen species (ROS)

Mitochondrial reactive oxygen species (ROS) formation was detected with 2, 7-dichlorofluorescein diacetate (DCFH2-DA), a fluorescent probe, according to the instruction of ROS assay kit (Beyotime Institute of Biotechnology, China) with a slight change. The MSCs were incubated with 10 μM DCFH2-DA dissolved in none-serum DMEM at 37°C for 20 min. The fluorescence was then measured at 488 nm excitation and 525 nm emission by FACS Calibur^™^ flow cytometer (BD Biosciences, San Jose, CA, U.S.)

### DAF-FM diacetate for nitric oxide (NO) indication

Viable cells were prepared in suspension. The DMSO stock solution was diluted into a suitable buffer with concentration of 10 μM. Cells were incubated with the diluted DAF-FM diacetate for 30 minutes at 37°C then were washed to remove excess probe. After replacing with fresh buffer, cells were incubated for an additional 30 minutes to allow complete de-esterification of the intracellular diacetates. Fluorescence excitation and emission maxima were 495 and 515 nm, respectively.

### Western blot

Following experimental treatments, cells were harvested, and lysed with lysis buffer. Protein concentration was measured with BCA protein assay reagent. The samples were diluted with lysis buffer, and equal amounts of protein were separated by SDS-PAGE. The separated proteins were transferred to polyvinylidene difluoride membranes. The membranes were incubated with various primary antibodies. After washing, the membranes were hybridized with horseradish peroxidase-conjugated secondary antibodies (Santa Cruz Biotechnology). The blots were detected using ECL Plus Western Blotting Substrate. The relative signal intensity of bands was determined and standardized. The primary antibodies used in the study include NF-κB p65 (dilution 1:1000, Cell Signaling Technology, Inc., Beverly, MA, USA), Phospho-NF-κB p65 (Ser536) (dilution 1:1000, Cell Signaling Technology, Inc., Beverly, MA, USA), IκBa (dilution 1:1000, Cell Signaling Technology, Inc., Beverly, MA, USA), Phospho-IκBa (dilution 1:1000, Cell Signaling Technology, Inc., Beverly, MA, USA). The same membrane was probed with anti-GAPDH (dilution 1:1000) as a loading control. Image J was used for analysis of intensity of bands in the Western blotting.

### Human lymphocytes cultures and phytohaemagglutinin (PHA) stimulation

Purified peripheral blood mononuclear cell (PBMC) was prepared with the help of centrifugation on Ficoll-Isopaque (Lymphoprep, Nycomed, Oslo, Norway) and was cultured in RPMI 1640 medium supplemented with HEPES (25mmol/l), penicillin (100 U/ml), streptomycin (100 μg/ml), L-glutamine (2mmol/l) (Gibco BRL) and 10% fetal bovine serum (Sigma, St Louis, MO, USA). MSC at 1 × 10^5^/ml density were inoculated per well into 24-well plate. After incubation for 2 h, group 1 as a control group without treatment; group 2 were treated with LPS (100 μg/ml) for 1 h, and group 3 were treated with LPS as group 2 plus LLL irradiated for 1 h. After adding 5 × 10^5^ mononuclear cells per well in 24-well plate to coculture with various types of MSCs or culture alone as control, 1ml RPMI1640 working fluid [1640 medium + PHA activator (50μg / ml) + penicillin (100 U/ml) and streptomycin (100 μg/ml) + 10% FBS] were added, then stay in the incubator for 24 hours.

### Flow cytometry

Detached cells were resuspended in PBS. Lymphocytes were stained with conjugated, monoclonal antibodies against CD25 or CD69, respectively. The cells were assayed in a flow cytometre (Accuri^™^ C6, Becton Dickinson) and the data were analyzed with CFlow Plus software (Becton Dickinson).

### Statistical analysis

Results were expressed as mean±SD of three independent experiments. All data were analyzed with SPSS 22.0 for Windows. Differences between two groups were assessed using unpaired two-tailed t-tests. Data involving more than two groups were assessed by analysis of variance (ANOVA) followed by a post hoc Tukey’s test for multiple comparisons. P values <0.05 were considered statistically significant.

## Results

### LLL changed inflammatory cytokine expression in MSCs

**Fig 1:**
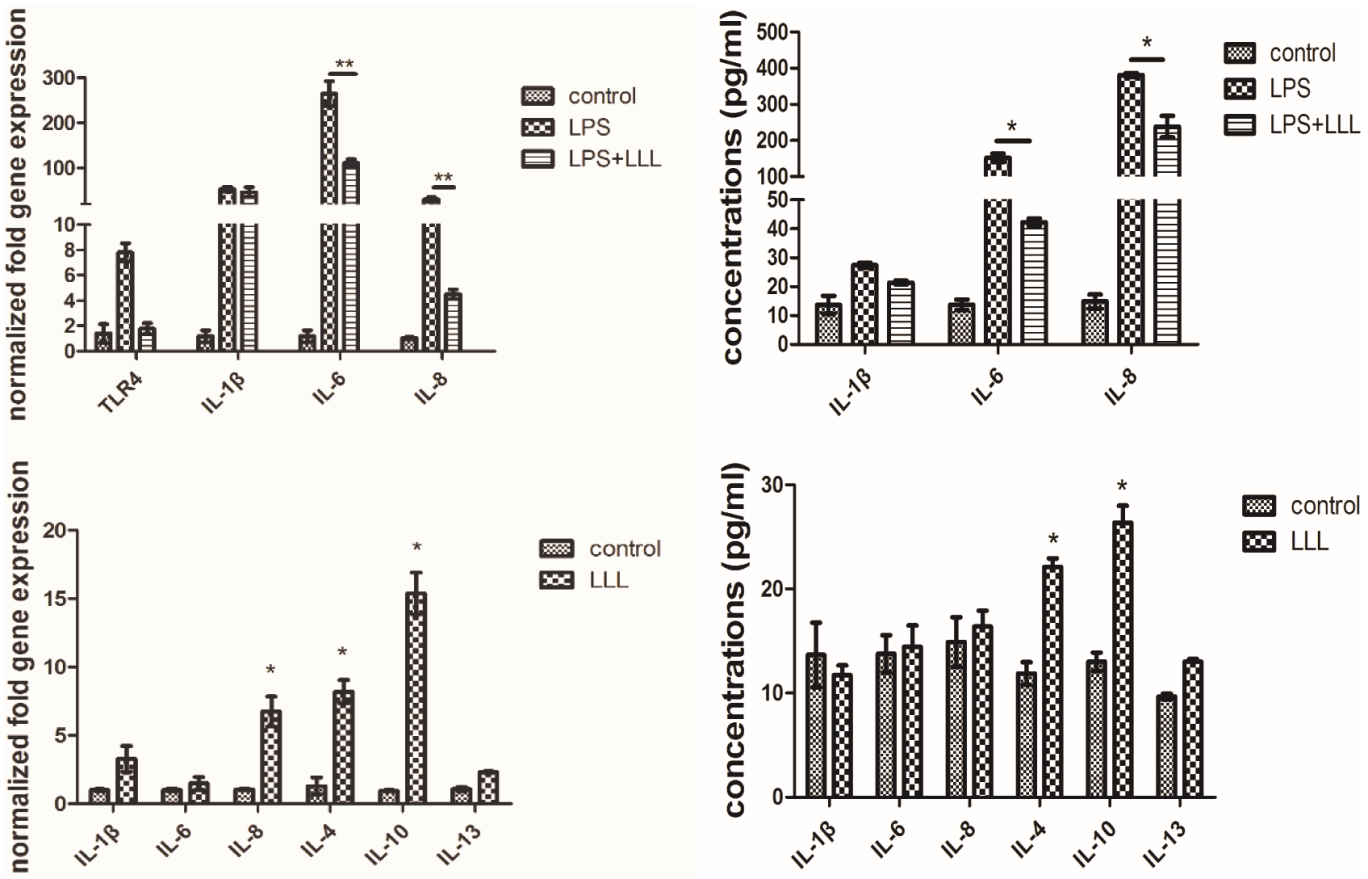
Effect of LLL on mRNA expression and production and of IL-1β, IL4, IL-6, IL-8 and IL-10 by LPS (or not) induced MSCs. MSCs were incubated with or without LPS (10ng/mL) and simultaneous treated LLL for 1h. Values of *P<0.05, **P<0.01and vs. LPS or control were considered statistically significant.

The secretion pattern of LPS-treated cells appears to favor proinflammatory mediators such as IL-1β, IL6, and IL8, ELISA confirmed that LPS treatment resulted in the increased secretion of IL-1β, IL6, and IL8. When MSCs were treated with a combination of LLL and LPS, LLL significantly reduced the levels of IL-6 and IL-8 mRNA expression compared to LPS treated alone. The concentration of IL-6 and IL-8 in culture medium was measured by ELISA, LLL combined with LPS significantly suppressed IL-6 and IL-8 production compared to LPS treated alone. TLR4 showed a higher mRNA expression level in LPS induced MSCs. MSCs irradiated by LLL increased IL8, IL-4 and IL-10 mRNA expression, IL-4 and IL-10 production also increased after treated with LLL measured by ELISA (Fig 1).

### LLL decrease nuclear translocation of NF-κB induced by LPS

**Fig 2:**
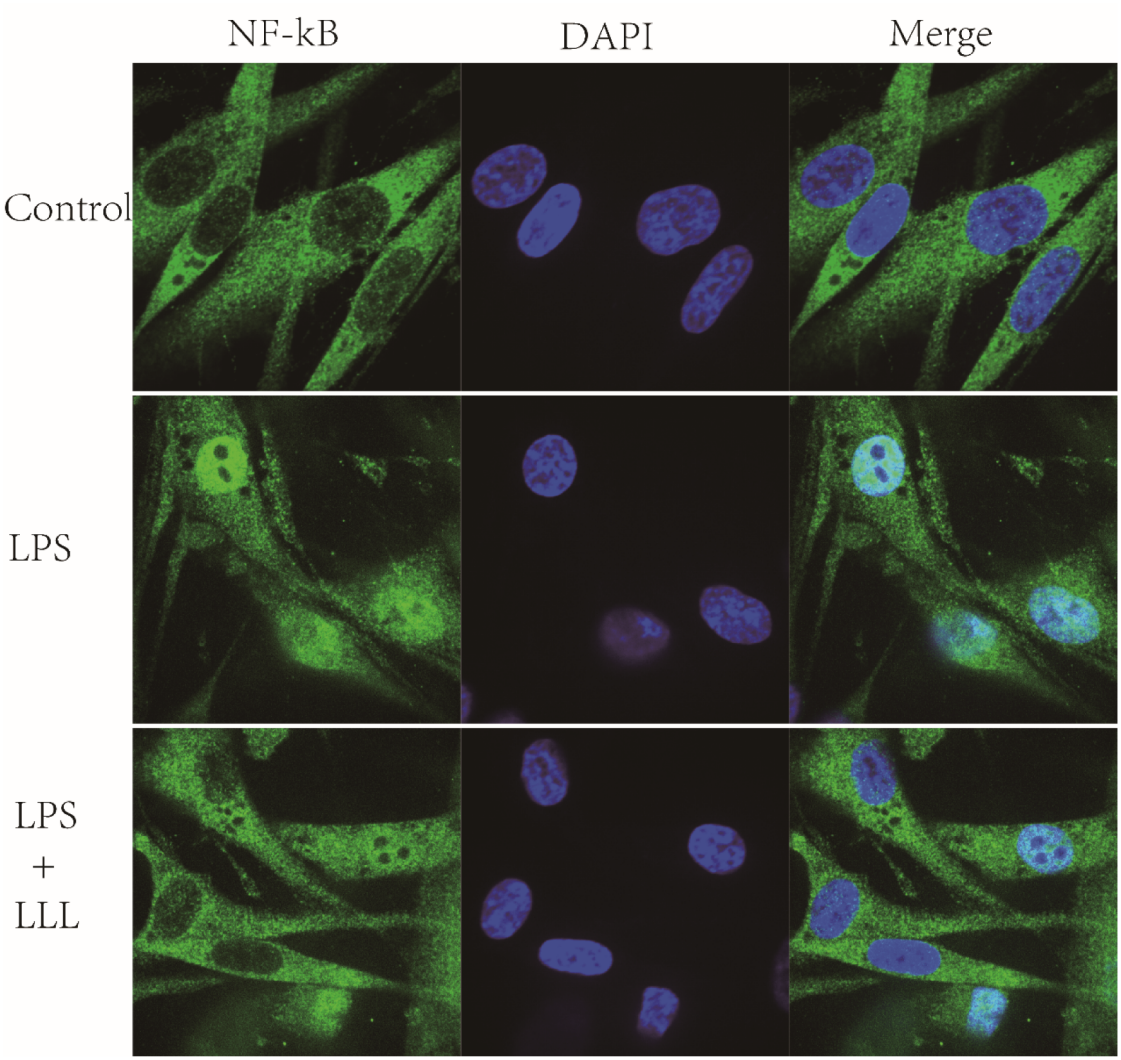
LLL decrease nuclear translocation of NF-κB. The images show the cytoplasmic localization of NF-κB in the control cells (upper panel), the nuclear translocation of NF-κB in cells treated with LPS (middle panel) and LLL treatment blocked the nuclear translocation of NF-κB caused by LPS stimulation (lower panel).

Stimulated NF-κB can induce its translocation into the nucleus where it binds to the promoter regions of several proinflammatory genes. We monitored the cellular distribution of NF-κB using fluorescence microscopy and found that treatment with LPS significantly increased NF-κB p65 translocation into the nucleus. However, in the presence of LLL irradiation, the elevation nuclear translocation of NF-κB was blocked in LPS-stimulated MSCs (Fig 2).

### LLL suppress phosphorylation of NF-κB p65 and IκBα induced by LPS in MSCs

**Fig 3:**
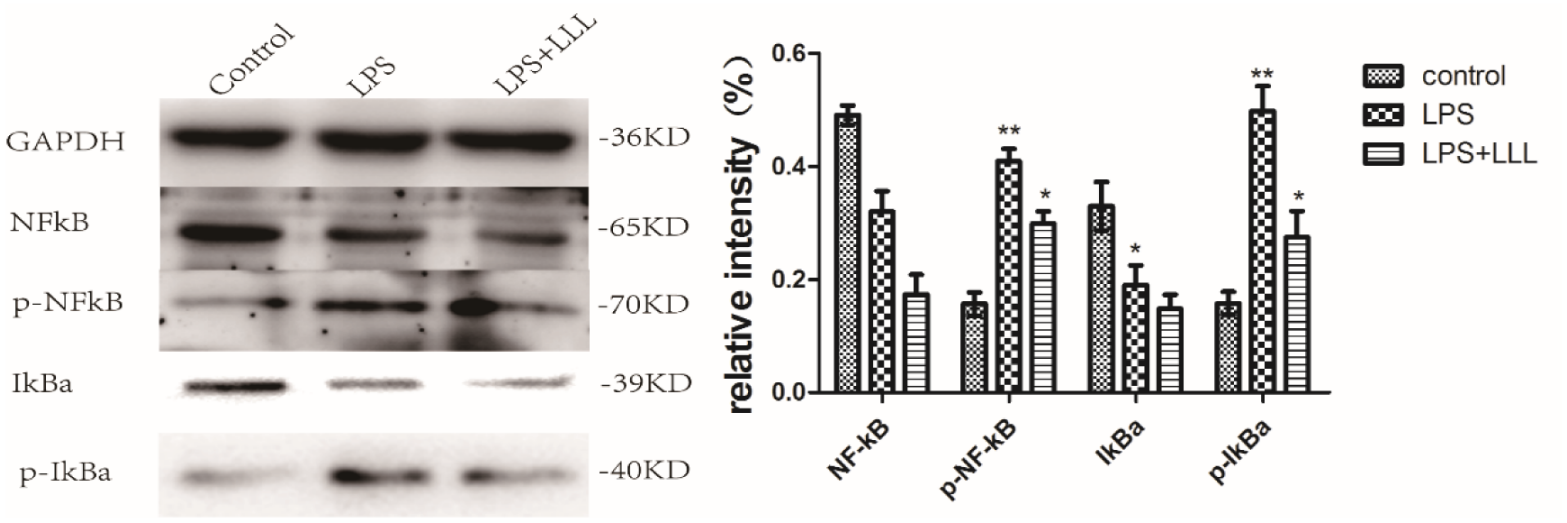
LLL suppress the LPS-induced activation of the NF-κB pathway in MSCs. Protein samples were analyzed by Western blot using anti p65, p-p65, IκBα and p-IκBα antibody (left), GAPDH was used as the internal control for normalization. The bar chart shows the quantitative evaluation of protein bands by densitometry (right). The data represent the mean±SD (n = 3 per group) *P<0.05, **P<0.01 vs. LPS or control were considered statistically significant.

TLRs within MSCs were stimulated for 1 hour by LPS (10 ng/mL) with or without LLL and assessed by Western blot analysis to examine phosphorylation of NF-κB p65 and IκBα. Treatment with LPS induced a 2-fold increase in p-NF-κB p65 phosphorylation and a 3-fold increase in p-IκBα, IκBa degradation was observed in LPS treated group. when MSCs were co-treated with LPS and LLL, the expression of p-NF-κB p65 and p-IκBα were inhibited compared to LPS treated alone (Fig 3).

### LLL downregulates LPS-induced ROS and NO production

**Fig 4:**
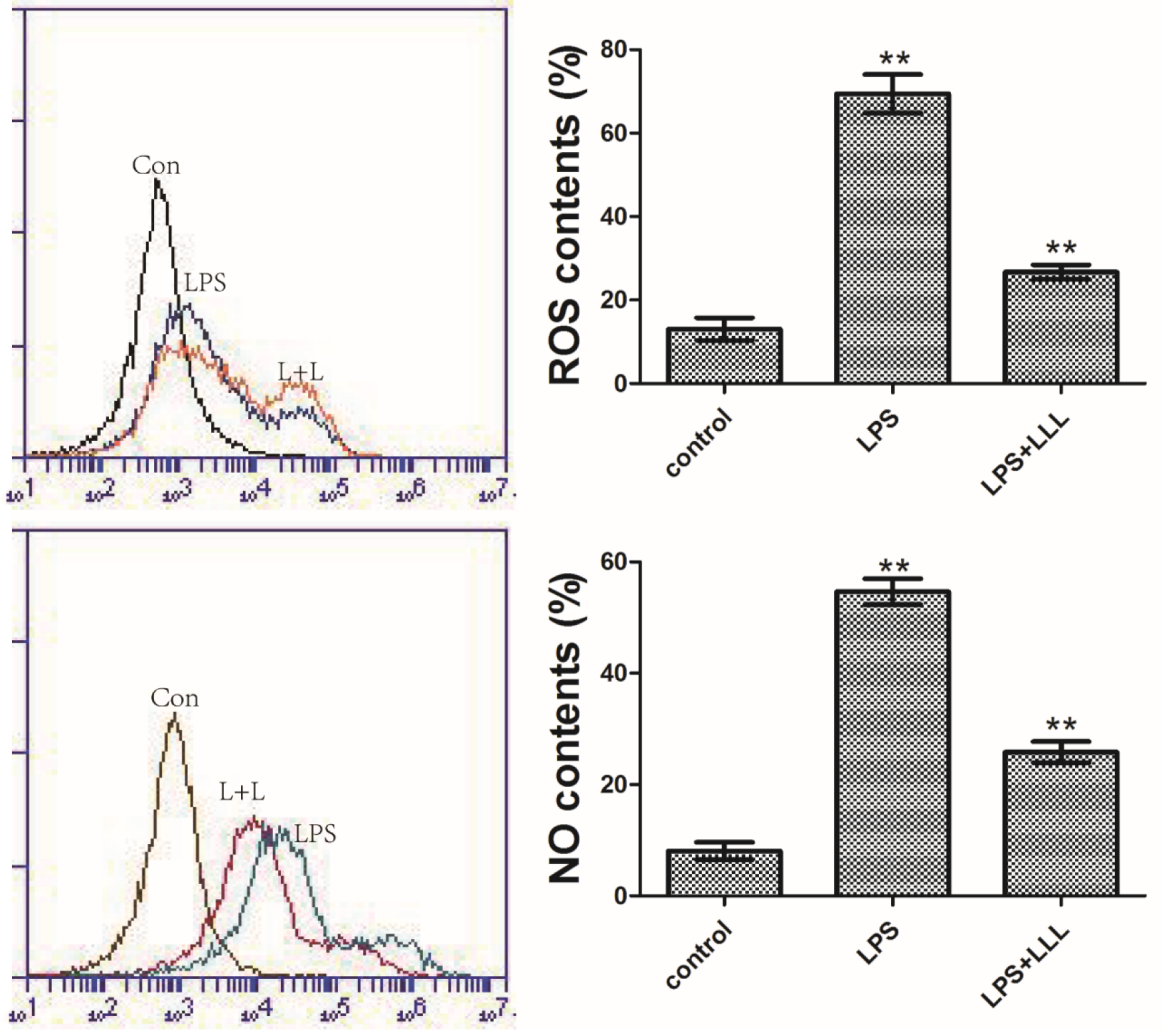
LLL suppress ROS and NO promotion induced by LPS in MSCs. MSCs were incubated with LPS (10ng/mL) and simultaneous treated with LLL for 1 h. Error bars represent the mean±SD (n = 3 per group). Values of **P<0.01 vs. LPS and **P<0.01 vs. control were considered statistically significant.

LLL stimuli alone increase the production of reactive oxygen species (ROS)[16]. ROS can also activate NF-κB that is accompanied with increased degradation of its inhibitor IκB[17]. The activated NF-κB, in turn, increases the expression of the iNOS and subsequent synthesis of NO[18]. LPS treatment triggered intensely overproduction of ROS and NO compared to that of the control group(**P<0.01). Upon LLL, the release of ROS induced by LPS is blocked and the release of NO induced by LPS is also blocked(**P<0.01) (Fig 4).

### PBMC (peripheral blood mononuclear cell) stimulation with PHA in the absence or presence of 3 types of MSCs

**Fig 5:**
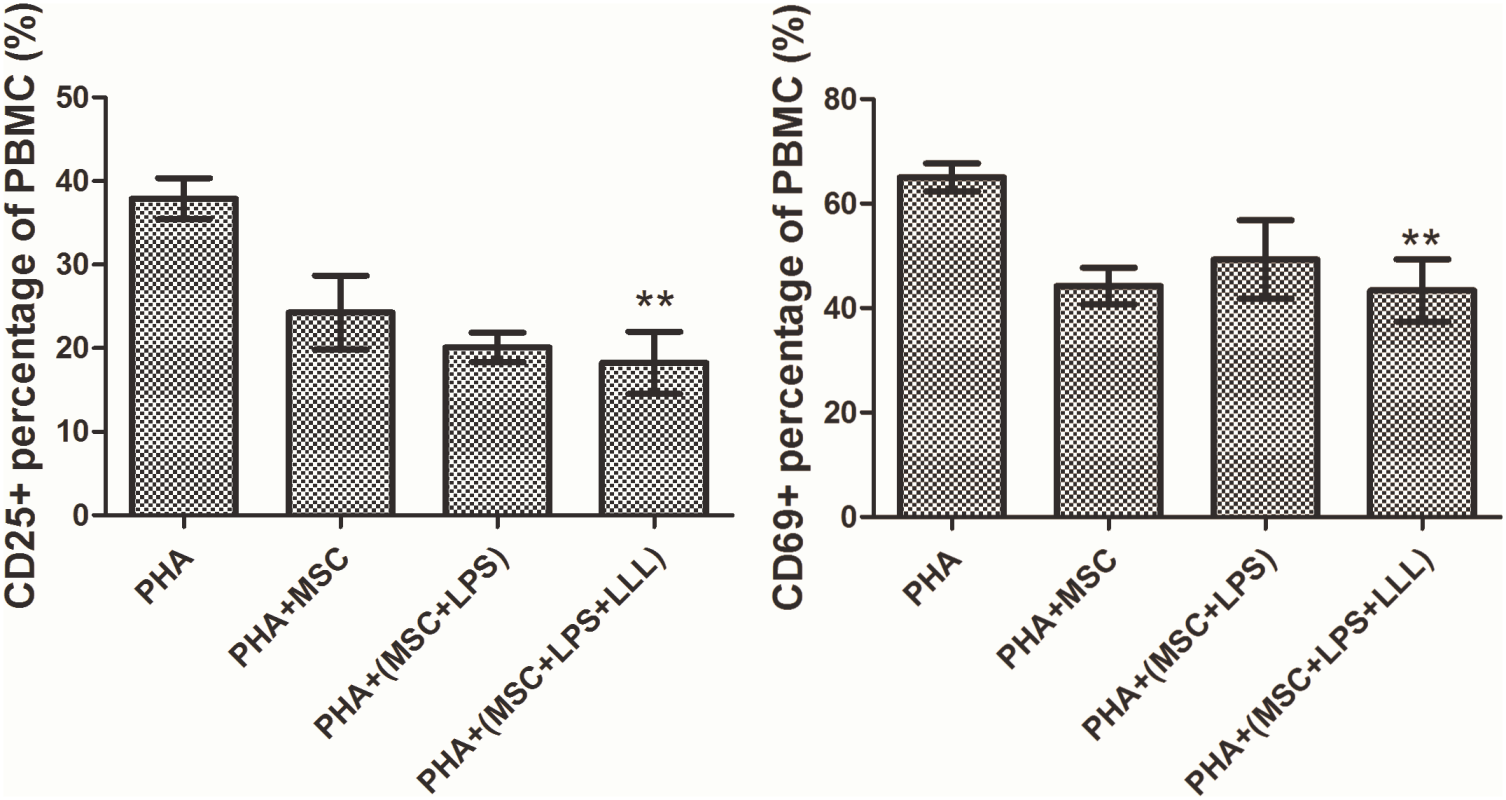
Percentage of positive PBMC after stimulation with PHA in the absence or presence of 3 types of treated MSCs. After reduction of LLL treated MSCs, CD25+ cells decreased (**P < 0.01 vs. PHA) and CD69+ cells were also reduced compared to control groups (**P < 0.01 vs. PHA).

Flow cytometric analysis of expression of cell surface antigens typically expressed by PHA-activated lymphocytes was performed. There was a report indicated that MSC significantly reduced the expression of activation markers CD25, CD38 and CD69 on PHA-stimulated lymphocytes[19]. In PBMC stimulated with PHA, we found decrease in the activation-associated markers CD25 and CD69 in co-cultures of PBMC and 3 types of treated MSCs (MSCs, LPS induced MSCs, LLL combined LPS induced MSCs). When LPS induced MSCs treated with LLL were present in the lymphocyte culture, the expression of CD25+ was significantly less than that in the controls (PHA treated only) (**P < 0.01), the early activation marker CD69 was also reduced (**P < 0.01), when MSC were present (Fig 5).

## Discussion

In this study, we confirmed the inhibitory effects of Low lever laser (LLL) on production and expression of inflammatory cytokines including IL-1β, IL-6, IL-8 in LPS-stimulated mesenchymal stem cells (MSCs), LLL treated alone increased the expression and secretion of anti-inflammatory cytokines such as IL-4 and IL-10 of MSCs. In addition, we have investigated to demonstrate the anti-inflammatory mechanism. LLL blocked the nuclear accumulation of NF-κB, decreased IκBa degradation and NF-κB activation induced by LPS stimulation. Intensely increased ROS generation and NO secretion after LPS stimulation were inhibited following combined treatment with LLL. LLL treated MSCs significantly inhibited the expression of CD25 and CD69 on co-cultured phytohaemagglutinin-activated PBMC.

It is known that MSCs are recruited to sites of stress or inflammation to fulfill their repair function. An emerging concept is that MSCs are not spontaneously immunosuppressive but require ‘licensing’ or activation to exert their immunosuppressive effects[20]. Recent reports indicate that mesenchymal stem/progenitor cells (MSCs) are among the cells that express TLR proteins and TLR signaling has been implicated in the licensing of MSCs [21–23]. Pro-inflammatory cytokines are a group of proteins secreted by MSCs after stimuli from environment of trauma, which play essential roles in regulating cell reaction[24]. With TLR4 priming inducing a pro-inflammatory phenotype and secretion of IL-6, IL-8 and TGF-b, in contrast, TLR3 priming induced anti-inflammatory MSCs (producing IDO, PGE-2, IL-4 and IL-1RA)[25]. Many of the findings have indicated that LLL suppresses the inflammatory reaction both in vitro and in vivo[26–28]. In LPS treated MSCs, we obtained similar mRNA expression and production of pro-inflammatory cytokines (TLR4, IL-1β, IL-6, and IL-8). Combining LPS treatment with LLL significantly inhibited IL-6 and IL-8 production, while LLL treated alone led an anti-inflammatory effect of production of IL-4 and IL10.

Many studies have indicated that LPS can trigger NF-κB signaling pathways via promoting phosphorylation of IκB-α and NF-κB p65. [29–31]. After stimulation by upstream signals, the IκB kinase (IKK) complex phosphorylates IκBs, leading to proteasome-mediated degradation and dissociation of IκBa and NF-κB phosphorylation, in the process nuclear translocation of NF-κB are required for NF-κB activation[32]. In LPS-induced model, the transcription of the iNOS, TNF-α, IL-lβ and IL-6 genes were directly regulated, since they all contain NF-κB binding sites[33]. LLL decreases the expression of LPS-induced proinflammatory cytokines by regulating NF-κB activity. Fluorescence microscopy indicated that LLL blocked the nuclear accumulation of NF-κB resulting from LPS stimulation. Western Blot analysis showed that LLL decreases IκBa degradation and NF-κB activation induced by LPS treatment.

The transcription of NF-κB-dependent genes influenced the levels of ROS in the cell, and in turn, the levels of NF-κB activity were also regulated by the levels of ROS[34]. ROS interacted with NF-κB at various places within the signaling pathway and often stimulated the NF-κB pathway in the cytoplasm, but inhibited NF-κB activity in the nucleus[35]. NF-κB activation also upregulated inducible nitric oxide synthase (iNOS) leading to enhanced nitric oxide (NO) production during an inflammatory response[36]. In our study, ROS generation and NO secretion were intensely increased after LPS stimulation. Compared with the LPS-alone activated group, the inhibitory rates appeared following combined treatment with LLL. The fact that the addition of LLL abrogated the activation of NF-κB provides additional evidence that ROS and NO were involved in the activation of NF-κB.

MSCs could act on all cells of the immune system, which include the capacity to inhibit the proliferation and function of T-cells[19] with mechanisms involve cell-cell contact, release of soluble factors, and generation of regulatory lymphocytes[37]. Study showed that the stimulation of TLR3 and TLR4 before the coculture with T-cells enhanced the immunomodulatory capacity of MSCs through the indirect induction of IDO1[38]. In the present study, we confirmed the inhibitory effect of MSC on PBMC proliferation triggered by PHA stimuli. An evaluation of activation associated markers (CD25^+^, CD69^+^) confirmed our findings. Our investigation has provided the evidence that treatment with TLR4-antagonist combined with LLL can significantly attenuate PBMC active responses.
